# Real-world overall survival with abiraterone acetate versus enzalutamide in chemotherapy-naïve patients with metastatic castration-resistant prostate cancer

**DOI:** 10.1038/s41391-024-00816-0

**Published:** 2024-03-27

**Authors:** Daniel J. George, Krishnan Ramaswamy, Hongbo Yang, Qing Liu, Adina Zhang, Alexandra Greatsinger, Jasmina Ivanova, Betty Thompson, Birol Emir, Agnes Hong, Stephen J. Freedland

**Affiliations:** 1 Duke Cancer Institute, Durham, NC USA; 2 Pfizer Inc., New York, NY USA; 3 Analysis Group, Inc., Boston, MA USA; 4 Formerly of Astellas Pharma, Northbrook, IL USA; 5 Cedars-Sinai Medical Center, Los Angeles, CA USA; 6 Durham VA Medical Center, Durham, NC USA

**Keywords:** Prostate cancer, Cancer therapy

## Abstract

**Background:**

There are no large head-to-head phase 3 clinical trials comparing overall survival (OS) for abiraterone and enzalutamide. This study used Medicare claims data to compare OS in patients with chemotherapy-naïve metastatic castration-resistant prostate cancer (mCRPC) who initiated abiraterone or enzalutamide.

**Methods:**

This retrospective analysis of the Medicare database (2009–2020) included adult men with ≥1 claim for prostate cancer, metastatic diagnosis, and no prior chemotherapy or novel hormone therapy who initiated first-line (1L) abiraterone or enzalutamide in the index period (September 10, 2014 to May 31, 2017). Cox proportional-hazards models with inverse probability treatment-weighting (IPTW) were used to compare OS between abiraterone- and enzalutamide-treated patients, adjusting for baseline characteristics. Subgroup analyses by baseline characteristics were also conducted.

**Results:**

Overall, 5506 patients who received 1L abiraterone (*n* = 2911) or enzalutamide (*n* = 2595) were included. Median follow-up was comparable in both cohorts (abiraterone, 19.1 months; enzalutamide, 20.3 months). IPTW-adjusted median OS (95% CI) was 20.6 months (19.7‒21.4) for abiraterone and 22.5 months (21.2‒23.8) for enzalutamide, with an IPTW-adjusted hazard ratio (95% CI) of 1.10 (1.04–1.16). Median OS was significantly shorter for abiraterone versus enzalutamide in patients ≥75 years old; White patients; patients with baseline diabetes, cardiovascular disease, both diabetes and cardiovascular disease, and renal disease; and across all socioeconomic strata.

**Conclusions:**

In the Medicare chemotherapy-naïve mCRPC population, 1L abiraterone was associated with worse OS versus enzalutamide in the overall population and among subgroups with older age and comorbidities, supporting findings from previous real-world studies and demonstrating a disparity in outcomes.

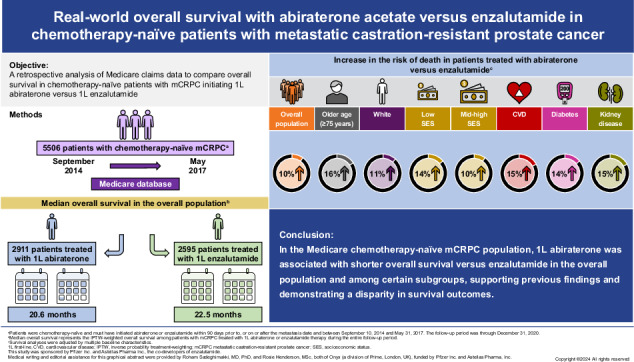

## Introduction

Prostate cancer (PC) is the most common cancer, excluding non-melanoma skin cancer, and the second leading cause of cancer death among men in the United States (US). In the COU-AA-302 (NCT00887198) and PREVAIL (NCT01212991) clinical trials, abiraterone and enzalutamide, respectively, showed clinically meaningful improvements in survival compared with placebo as first-line (1L) treatments in patients with metastatic castration-resistant PC (mCRPC) [1, 2]. As such, US guidelines recommend continued androgen deprivation therapy (ADT) in combination with either abiraterone plus prednisone or enzalutamide as the preferred novel hormone therapies (NHTs) for the treatment of NHT-naïve mCRPC [3]. Abiraterone is an androgen biosynthesis inhibitor, that inhibits 17α-hydroxylase/C17,20-lyase (CYP17), while enzalutamide is an androgen receptor inhibitor. There are no large head-to-head phase 3 clinical trials comparing the efficacy of abiraterone to enzalutamide as 1L treatment for mCRPC, with overall survival (OS) as the primary endpoint.

Several meta-analyses of clinical trials have demonstrated worse radiographic progression-free survival in patients with mCRPC following 1L treatment with abiraterone versus enzalutamide [4–6]. However, clear evidence of OS differences is not available from these meta-analyses and the reported findings are limited by the considerable heterogeneity in clinical trial populations and designs, and the reliance on immature OS data at the time of primary endpoint readout in some studies.

Multiple real-world studies have compared the OS associated with 1L abiraterone versus enzalutamide in patients with mCRPC. Six large studies (each >1000 patients), using data from the US Veteran’s Health Administration (VHA) [7, 8], US Flatiron electronic medical record (EMR) [9], French National Health System [10], and Taiwan National Health Insurance databases [11, 12], demonstrated statistically significant detriment in OS for abiraterone compared with enzalutamide in this population. Among studies with smaller sample sizes (100‒1000 patients), one reported a significantly reduced OS with abiraterone versus enzalutamide [13] and eight found comparable OS for 1L abiraterone and enzalutamide [14–21]. Of note, a non-significant trend for worse survival with abiraterone versus enzalutamide was observed in five of these studies [14, 17, 18, 20, 21]. Importantly, no study to date has shown improved OS for abiraterone versus enzalutamide.

Given the observed OS differences for 1L abiraterone versus enzalutamide in mCRPC studies using French and Taiwanese national datasets, it is valuable to examine these survival differences in a US national dataset, such as Medicare, which is more broadly representative of the population than the US VHA and US Flatiron EMR datasets. Medicare is the primary insurer for men aged ≥65 years in the US and, as the majority (~88%) of the deaths from PC occur in this age group, with a median age at death of 79 years, it is important to assess survival differences within this population [22, 23].

This study aimed to compare OS in chemotherapy-naïve patients with mCRPC initiating 1L abiraterone versus enzalutamide in the US Medicare population. Based on multiple prior real-world studies, we hypothesized that OS would be worse in abiraterone-treated patients relative to enzalutamide-treated patients.

## Methods

### Data source

This was a retrospective, observational study of administrative claims data from the Centers for Medicare and Medicaid Services 100% Medicare fee-for-service database from January 1, 2009 to December 31, 2020. Medicare is a US national program that provides access to health insurance for Americans aged ≥65 years, certain disabled patients aged <65 years, and patients with end-stage renal disease [24]. This study was determined to be exempt from review by the New England Institutional Review Board.

### Patient identification

Patients who were ≥18 years of age with ≥1 medical claim with a PC diagnosis code (ICD-9-CM: 185; ICD-10-CM: C61); metastatic disease; evidence of surgical castration any time before the index date or medical castration lasting ≥8 weeks within 1 year before index date; and a post-castration prescription claim for abiraterone or enzalutamide were included in the study (Fig. S1).

Patients were chemotherapy-naïve and must have initiated abiraterone or enzalutamide within 90 days prior to the metastasis date, or on or after the metastasis date and between September 10, 2014 and May 31, 2017 to ensure that both therapies were approved for chemotherapy-naïve mCRPC and prior to disclosure of clinical trial data for abiraterone use in metastatic castration-sensitive PC (mCSPC). The index date was defined as the initiation date of abiraterone or enzalutamide. The start of the index period was based on the date of the US Food and Drug Administration (FDA) approval of enzalutamide for chemotherapy-naïve mCRPC. Abiraterone was approved for use in chemotherapy-naïve mCRPC in 2012. Enzalutamide was approved for use in chemotherapy-naïve mCRPC in September 2014. The end of the index period was shortly before the public disclosure of the clinical trial data on abiraterone efficacy in mCSPC [25], and was selected to ensure patients with mCSPC were excluded.

Patients were included in two distinct cohorts of abiraterone- and enzalutamide-treated patients based on their index prescription, using an intention-to-treat study design.

### Patient characteristics

Demographic characteristics (e.g., age, race, region, and socioeconomic status [SES]), clinical characteristics, comorbidities during the baseline period, prior treatments, and healthcare resource use were assessed on or within the 365 days prior to the index date.

Definitions for baseline characteristics and administrative codes for defining comorbidities are presented in the Supplementary Information and Table S1.

### Outcome measures

Length of follow-up, treatment patterns and duration, and OS were assessed from the index date to the earliest of death, disenrollment from Medicare, or the end of data availability.

OS was defined as the time from the index date to death from any cause. Treatment duration of the index prescription was defined as the time from the index date to the discontinuation date. Discontinuation was defined as the earliest of: (1) death, (2) last observed administration plus day of supply associated with last administration, or (3) day before the start of next line of therapy (LOT). Time to subsequent therapy was defined as the time from the index date to the start of next LOT.

### Statistical analysis

Means and standard deviations were estimated for continuous baseline variables. Counts and percentages were estimated for categorical baseline variables. Standardized mean difference (SMD) was calculated for each baseline variable. Treatment sequences of up to three treatment regimens were reported in Sankey diagrams. Kaplan–Meier analyses were conducted to describe time-to-event outcomes (i.e., OS, treatment duration, and time to subsequent treatment). Unadjusted and inverse probability treatment-weighting (IPTW)-adjusted Cox proportional-hazards models adjusting for baseline characteristics were fitted to compare time-to-event outcomes in the overall study population as well as in subgroups (Supplementary Information). Hazard ratios (HRs) with their 95% confidence intervals (CIs) and *p*-values were estimated from the Cox models using enzalutamide as the reference cohort.

### Sensitivity analysis

A sensitivity analysis was conducted to test the robustness of the primary analysis by adjusting the logistic regression model for additional covariates (Supplementary Information).

### Subgroup analysis by baseline characteristics

Subgroup analyses were conducted to compare OS between abiraterone and enzalutamide, defined by baseline characteristics: age (≥75 years, <75 years), race (White, Black), SES (low, middle/high), presence of comorbidities (cardiovascular disease [CVD], diabetes, liver disease, and renal disease).

### Subgroup analysis by subsequent treatment

Subgroup analysis was conducted to compare median OS between abiraterone and enzalutamide in patients who received 1L treatment with abiraterone or enzalutamide without any subsequent treatment. Additional exploratory analyses were conducted in the following subgroups: (1) patients who switched from abiraterone to enzalutamide and vice versa; (2) patients who switched from abiraterone or enzalutamide to chemotherapy; (3) patients who switched from abiraterone or enzalutamide to another LOT, i.e., non-NHT and non-chemotherapy second-line (2L) regimens, or received >2 LOTs.

## Results

### Patient population

Overall, 5506 patients with chemotherapy-naïve mCRPC who initiated 1L abiraterone or enzalutamide were identified: 2911 in the abiraterone cohort and 2595 in the enzalutamide cohort (Fig. S1).

Baseline demographic and clinical characteristics were generally similar between the cohorts (Table 1; Table S2). Patients in the abiraterone cohort had a higher use of long-term corticosteroids during the baseline period than patients in the enzalutamide cohort (14.7% vs. 7.9%, SMD = 21.5%). Individual relevant comorbidities were largely similar; however, baseline diabetes was less common in the abiraterone cohort than in the enzalutamide cohort (31.5% vs. 36.8%, SMD = −11.1%).

**Table 1:** Baseline demographics and clinical characteristics.

	Treatment cohort		
	Abiraterone (*n* = 2911)	Enzalutamide (*n* = 2595)	Standardized mean difference^a^ (%)
**Demographics at index date**^b^			
Age at the index date (years), mean ± SD	78.4 ± 7.9	78.6 ± 8.2	−3.29
Race, *n* (%)			
White, non-Hispanic	2273 (78.1)	2012 (77.5)	1.32
Black	364 (12.5)	336 (12.9)	−1.33
Hispanic	148 (5.1)	134 (5.2)	−0.36
Other/unknown^c^	126 (4.3)	113 (4.4)	−0.13
Low SES, *n* (%)	641 (22.0)	599 (23.1)	−2.54
Dual eligibility with Medicaid^d^	565 (19.4)	530 (20.4)	−2.54
Medicare Part D eligibility for low-income subsidy^e^	639 (22.0)	599 (23.1)	−2.71
**Clinical characteristics**			
Medication and procedure history^f^, *n* (%)			
Long-term corticosteroids^g^	428 (14.7)	206 (7.9)	21.47
First-generation antiandrogens	2017 (69.3)	1731 (66.7)	5.54
Ketoconazole	144 (4.9)	100 (3.9)	5.33
Radical prostatectomy	38 (1.3)	38 (1.5)	−1.36
Site of metastatic disease diagnosis, *n* (%)			
Bone (no viscera or node)	1861 (63.9)	1715 (66.1)	−4.53
Node (no bone or viscera)	122 (4.2)	132 (5.1)	−4.26
Bone and nodes (no viscera)	345 (11.9)	272 (10.5)	4.35
Viscera	519 (17.8)	395 (15.2)	6.93
Liver	113 (3.9)	85 (3.3)	3.26
Other	64 (2.2)	81 (3.1)	−5.74
Modified CCI (excluding cancer)^h^, mean ± SD	2.7 ± 2.5	2.8 ± 2.6	−4.92
Individual comorbidities^i^, *n* (%)			
Hypertension	2290 (78.7)	2105 (81.1)	−6.12
Hyperlipidemia	1935 (66.5)	1742 (67.1)	−1.40
Anemia	1181 (40.6)	1041 (40.1)	0.93
Type II diabetes	891 (30.6)	936 (36.1)	−11.60
Urinary tract infection	940 (32.3)	877 (33.8)	−3.20
Peripheral vascular disease	739 (25.4)	705 (27.2)	−4.05
Chronic pulmonary disease	699 (24.0)	640 (24.7)	−1.52
Renal disease	726 (24.9)	606 (23.4)	3.71
Chronic obstructive pulmonary disease	569 (19.5)	535 (20.6)	−2.67
Congestive heart failure	543 (18.7)	533 (20.5)	−4.75

*CCI* Charlson Comorbidity Index, *ICD* International Classification of Diseases, *NCI* National Cancer Institute, *SD* standard deviation, *SES* socioeconomic status.

^a^The standardized mean difference was multiplied by 100 to get the percent standardized mean difference. A value >10% or <–10% is considered a significant imbalance.

^b^The index date was defined as the first initiation of abiraterone or enzalutamide within 90 days prior to or any time after a metastatic disease diagnosis following prostate cancer diagnosis, and during the index period of September 10, 2014 through May 31, 2017.

^c^Other race includes Asian, Native American native, and other not further specified in Medicare.

^d^Any 1-month period where a patient was dually eligible for Medicaid on the index date was counted.

^e^Any 1-month period where a patient was eligible for Part D low-income subsidy on the index date was counted.

^f^Assessed during the baseline period (12-months period prior to index date).

^g^Long-term corticosteroid use was defined as the following: (1) treatment duration of ≥90 days with a gap of ≤30 days between consecutive pharmacy claims or, (2) at least two corticosteroid procedure claims (per Part B data) with at least 90 days apart during the baseline period.

^h^Modified CCI (excluding cancer) score was calculated using the NCI Comorbidity Index codes for defining comorbidities in ICD-9-CM and ICD-10 administrative data.

^i^Individual comorbidities with a prevalence of >20% in the abiraterone or enzalutamide cohorts are shown. Additional data are available in Table S2.

### Treatment duration

Median (95% CI) treatment duration was numerically shorter for the abiraterone cohort (6.7 [6.3‒7.0] months) compared with the enzalutamide cohort (7.4 [7.0‒7.9] months), but the HR was not statistically significant (IPTW-adjusted HR 1.04 [95% CI 0.99–1.10]). In contrast, the median (95% CI) time to subsequent treatment was significantly shorter for the abiraterone cohort (14.5 [13.4‒15.4] months) compared with the enzalutamide cohort (16.7 [15.9‒17.8] months; IPTW-adjusted HR 1.14 [95% CI 1.06–1.22]; *p* < 0.001).

### Overall survival in the overall population

**Fig. 1:**
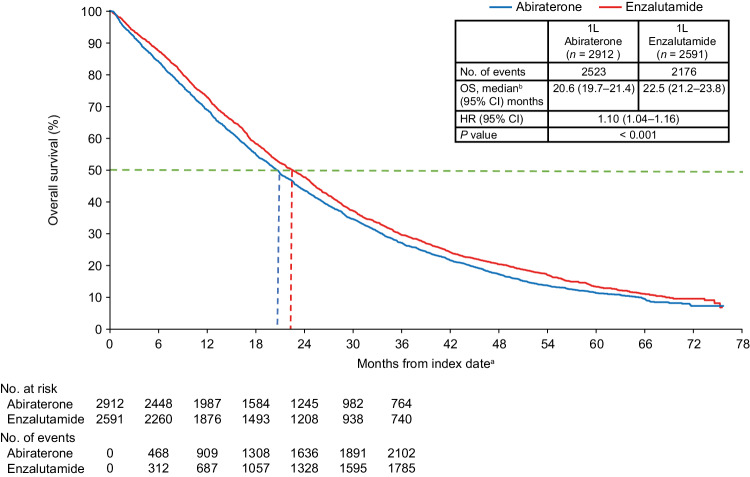
IPTW-adjusted pairwise OS comparison in the overall population of patients with chemotherapy- and NHT-naïve mCRPC. 1L first-line, ADT androgen deprivation therapy, CCI Charlson Comorbidity Index, CI confidence interval, HR hazard ratio, IPTW inverse probability treatment-weighting, mCRPC metastatic castration-resistant prostate cancer, NHT novel hormone therapy, OS overall survival, PC prostate cancer, SES socioeconomic status. ^a^The index date was defined as the first initiation of abiraterone or enzalutamide within 90 days prior to or any time after a metastatic disease diagnosis following PC diagnosis, and during the index period of September 10, 2014 through May 31, 2017. ^b^Median OS represents the IPTW-weighted OS among patients with mCRPC treated with 1L abiraterone or enzalutamide therapy during the entire follow-up period. Propensity scores for IPTW were generated by adjusting for baseline characteristics including age, race, geographic regions, SES, site of metastasis, liver metastasis, time from diagnosis to metastasis, time from metastasis to index date, time from ADT start to index date, radical prostatectomy, prior first-generation antiandrogens, prior chronic corticosteroid use, opioid analgesic use, comorbidities during baseline (CCI components, type I and type II diabetes, cardiovascular disease, and anemia), PC-related hospitalization, PC-related emergency room visit, all-cause hospitalization, and all-cause emergency room visits.

Median follow-up was similar for both cohorts (abiraterone: 19.1 months; enzalutamide: 20.3 months). The IPTW-adjusted median OS was 20.6 months for the abiraterone cohort and 22.5 months for the enzalutamide cohort. Patients who received abiraterone at index had an increased risk of death compared with patients who received enzalutamide (IPTW-adjusted HR 1.10; 95% CI: 1.04–1.16; *p* < 0.001) (Fig. 1).

In the sensitivity analysis using additional covariate adjustments, the results were identical to the main analysis (Fig. S2).

### Overall survival in subgroups defined by baseline characteristics

**Fig. 2:**
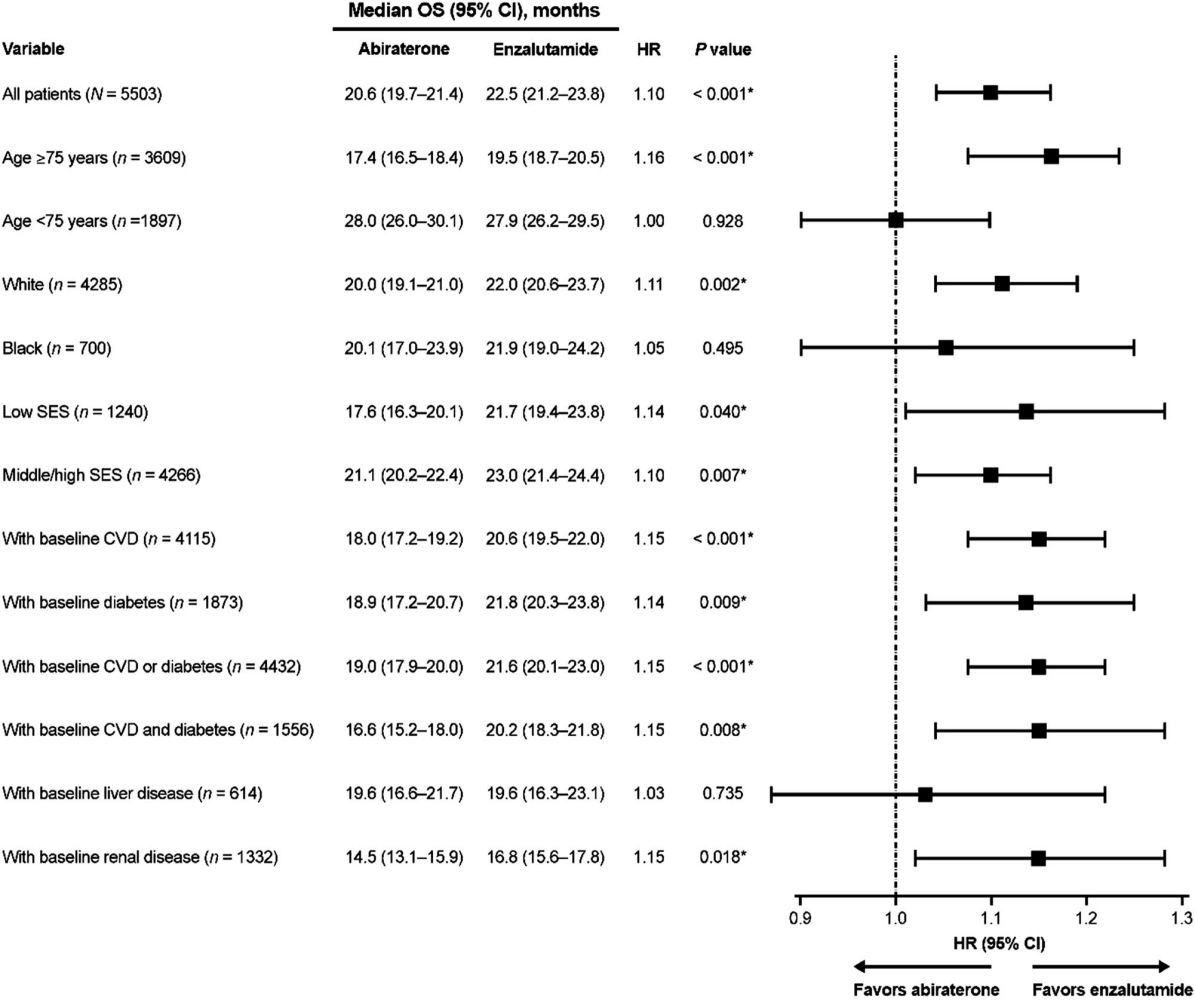
IPTW-adjusted pairwise OS comparisons among patients with mCRPC by subgroups of interest. *Indicates a statistically significant association. CI confidence interval, CVD cardiovascular disease, HR hazard ratio, IPTW inverse probability treatment-weighting, mCRPC metastatic castration-resistant prostate cancer, OS overall survival, SES socioeconomic status.

Shorter survival for abiraterone-treated patients versus enzalutamide-treated patients was found for multiple subgroups defined by baseline characteristics (Fig. 2). Worse survival for abiraterone versus enzalutamide was observed in patients ≥75 years old (HR 1.16, *p* < 0.001), White patients (HR 1.11, *p* = 0.002), patients with low and middle/high SES (HR 1.14, *p* = 0.040; and HR 1.10, *p* = 0.007, respectively), patients with baseline CVD (HR 1.15, *p* < 0.001), diabetes (HR 1.14, *p* = 0.009), both CVD and diabetes (HR 1.15, *p* = 0.008), and renal disease (HR 1.15, *p* = 0.018). There was no difference in OS between abiraterone and enzalutamide for Black patients and patients aged <75 years.

### Overall survival in subgroups defined by subsequent treatments

IPTW-adjusted median OS and IPTW-adjusted HRs for the subgroups defined by subsequent treatments are reported in Table 2. While OS was generally shorter for abiraterone compared with enzalutamide when stratified by subsequent treatment, this did not always reach statistical significance.

**Table 2:** IPTW-weighted pairwise OS comparisons among patients with mCRPC by treatment sequence subgroups.

Treatment sequence	Treatment cohort				Adjusted HR (95% CI)	*P* value
	Abiraterone (*n* = 2912)		Enzalutamide (*n* = 2591)			
	*n* (%)^a^	Median OS (95% CI), months	*n* (%)^a^	Median OS (95% CI), months		
Patients receiving 1L NHT only	1168 (40)	10.6 (9.6–11.7)	1075 (41)	13.6 (12.4–15.1)	1.16 (1.06–1.27)	0.001^*^
Patients with crossover to other NHT only	523 (18)	18.0 (16.7–20.6)	360 (14)	20.4 (18.6–23.4)	1.08 (0.93–1.25)	0.304
Patients switching to 2L chemotherapy only	138 (5)	14.6 (12.4–16.2)	115 (4)	14.0 (12.2–18.4)	1.11 (0.85–1.43)	0.433
Patients switching to other subsequent regimens^b^	1082 (37)	31.3 (29.7–33.1)	1042 (40)	31.5 (29.8–33.5)	1.05 (0.96–1.15)	0.296

1L first-line, *2L* second-line, *CI* confidence interval, *HR* hazard ratio, *IPTW* inverse probability treatment-weighting, *mCRPC* metastatic castration-resistant prostate cancer, *NHT* novel hormone therapy, *OS* overall survival.

^*^Indicates a statistically significant association.

^a^Sample sizes and percentages corresponding to each treatment sequence represent the number and proportion of patients in each treatment cohort per treatment sequence subgroup after IPTW adjustment.

^b^Patients in this subgroup included those who received 2L non-chemotherapy and non-NHT treatments and those who received more than one additional therapy post the index treatment.

### Treatment patterns

**Fig. 3:**
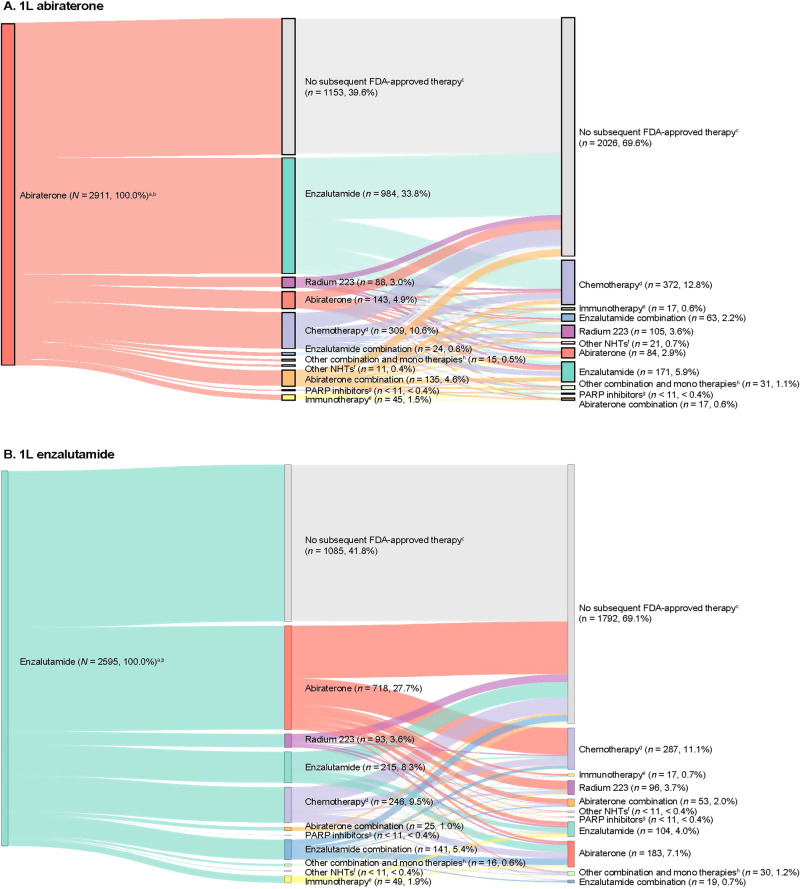
Treatment patterns among patients with mCRPC initiating 1L abiraterone or enzalutamide. **A** Patients who initiated abiraterone as 1L treatment and **B** patients who initiated enzalutamide as 1L treatment. 1L first-line, 2L second-line, 3L third-line, ADT androgen deprivation therapy, FDA US Food and Drug Administration, LOT line of therapy, mCRPC metastatic castration-resistant prostate cancer, NHT novel hormonal therapy, PARP poly (adenosine diphosphate ribose) polymerase. ^a^Percentages are reported out of the total number of patients. ^b^2L and 3L indicate treatment regimens after abiraterone (**A**) or enzalutamide (**B**), which were assessed during the follow-up period. ^c^No new subsequent FDA-approved therapy was defined as patients who did not receive NHTs, chemotherapy, immunotherapy, radium-223, ketoconazole, or PARP inhibitors as a subsequent line additional to ADT or older first-generation antiandrogens following their index treatment. ^d^Chemotherapy includes cabazitaxel, docetaxel, carboplatin, cisplatin, oxaliplatin, and mitoxantrone. ^e^Immunotherapy includes sipuleucel-T and pembrolizumab. ^f^Other NHTs include apalutamide and darolutamide. ^g^PARP inhibitors include olaparib and rucaparib. ^h^Other combinations and monotherapies include cabazitaxel + carboplatin, carboplatin + docetaxel, docetaxel + cisplatin, cabazitaxel + docetaxel, carboplatin + olaparib, cisplatin + docetaxel, docetaxel + radium-223, ketoconazole + docetaxel, ketoconazole + sipuleucel-T, mitoxantrone + carboplatin, sipuleucel-T + radium-223, ketoconazole, etc.

Comparable proportions of patients in the abiraterone (*n* = 1758, 60.4%) and enzalutamide (*n* = 1510, 58.2%) cohorts received at least one new subsequent FDA-approved therapy in addition to ADT or older first-generation antiandrogens following their index treatment (Fig. 3). Among the 2238 patients who did not receive a subsequent therapy, 13.0% continued on 1L treatment (abiraterone or enzalutamide), 66.4% discontinued 1L treatment, and 20.6% died on 1L treatment.

The most common 2L therapy for patients who received abiraterone at index was enzalutamide (*n* = 984, 33.8%), and abiraterone for patients who received enzalutamide at index (*n* = 718, 27.7%; Fig. 3). Among patients who received abiraterone or enzalutamide at index and received 2L therapy, 50.3% (*n* = 885) and 53.2% (*n* = 803) received a third line (3L) of therapy, respectively. Chemotherapy was the most common 3L therapy.

## Discussion

To our knowledge, this is the first retrospective real-world analysis assessing OS for abiraterone versus enzalutamide in mCRPC using the Medicare database. This study demonstrated worse survival associated with abiraterone compared with enzalutamide in an older population of patients with chemotherapy-naïve mCRPC (mean age of 78 years), which is largely representative of the US population at risk. Medicare represents a broad-based elderly US population for which survival differences may reflect both the efficacy and the tolerability of treatment. The 10% decrement in survival associated with abiraterone relative to enzalutamide reported here, in this frail population, is clinically significant and may have important implications for clinicians deciding between these treatment options that have identical indications for patients with mCRPC. Indeed, our subgroup analyses suggest that in patients with cardiovascular and metabolic comorbidities, treatment with abiraterone is associated with an even shorter proportional survival than treatment with enzalutamide.

The results of this analysis suggesting worse OS associated with abiraterone versus enzalutamide in the mCRPC population (HR 1.10, 95% CI: 1.04–1.16) are in line with previous analyses of large real-world studies using data from administrative claims and EMR [7–12]. In a study using the French National Health Data System (*N* = 10308; 2014‒2018), abiraterone was associated with shorter median OS compared with enzalutamide (31.7 vs. 34.2 months) [10]. Likewise, two retrospective analyses of the Taiwan National Health Insurance data (each >1000 patients) found significantly lower propensity-score-adjusted OS rates for 1L abiraterone versus 1L enzalutamide in the overall mCRPC population (49.51% vs. 57.6%, *p* = 0.003; and 46.3% vs. 59.4%, *p* < 0.001, respectively) [11, 12].

Real-world studies in selected US populations have reported similar OS differences. In a study using the US VHA database (2014‒2018) in chemotherapy-naïve patients with mCRPC (*N* = 3174), median OS was shorter with 1L abiraterone versus 1L enzalutamide (25.9 vs. 29.6 months, *p* = 0.001) [7]. Another VHA-based real-world study (*N* = 5822; 2014‒2017) also found significantly shorter median OS for abiraterone-treated patients compared with enzalutamide-treated patients (22.1 vs. 24.2 months, *p* = 0.001) [8]. Furthermore, a retrospective cohort study of mCRPC patients who received 1L systemic therapy using the US Flatiron EMR database (2012–2018) found shorter median OS for abiraterone versus enzalutamide among 2615 non-Hispanic White men (17 vs. 20 months; adjusted HR 1.21, 95% CI: 1.06‒1.38) [9]. Together, these results present robust evidence to support a reduced OS associated with 1L abiraterone compared with 1L enzalutamide in chemotherapy-naïve mCRPC patients.

In contrast to these large real-world studies, institutional cohort and population-based studies have reported comparable OS among mCRPC patients receiving 1L abiraterone or enzalutamide, although there was a non-significant trend favoring enzalutamide in several studies [14, 15, 17, 18, 20, 21, 26, 27]. However, these studies were smaller in size and underpowered to detect modest differences. Ultimately, Medicare represents the broadest real-world population database of patients with advanced PC in the US. Thus, it is critically important to determine if outcomes differ between these two NHTs as the most common standard-of-care options for patients with mCRPC.

Importantly, the threshold for tolerance of inferior outcomes in subgroups of patients undergoing therapeutic interventions in advanced cancers has come under increasing scrutiny [28]. In this study, significant abiraterone-associated detriments in OS persisted in several subgroups defined by baseline characteristics, including patients aged ≥75 years and those with CVD, diabetes, or both. This is supported by Schoen et al. who reported significantly shorter OS in patients aged ≥75 years and patients with CVD or diabetes who received abiraterone versus enzalutamide in VHA medical facilities [8]. The finding of poorer OS in older patients or patients with CVD treated with abiraterone versus enzalutamide, if causal, may stem from the greater cardiovascular toxicity associated with abiraterone plus prednisone relative to enzalutamide [29–32]. This may also explain the finding of poorer OS with abiraterone versus enzalutamide in the overall study population, as the majority of patients in this study were older and had baseline CVD. Importantly, we found no subgroups in which abiraterone was associated with an improvement in OS compared with enzalutamide.

Consistent with the findings of the Flatiron study by Marar et al. [9], our analysis by race demonstrated reduced OS with 1L abiraterone versus 1L enzalutamide in White patients, but no difference in Black patients. A limitation of both the current study and the Flatiron study [9], is that the proportion of Black patients included was low (13%). Other studies suggest that there may be a race-treatment effect, with a lower risk of death for Black patients versus White patients with mCRPC receiving 1L NHT [33, 34], but small sample sizes of Black patients limit the power for definitive conclusions at this time. Of note, unlike the current study, where we found no differences in OS for Black patients receiving abiraterone or enzalutamide, a recent analysis of the VHA dataset (2011–2017) that included a larger proportion of Black patients with mCRPC (23%) found significantly shorter OS associated with 1L abiraterone versus enzalutamide among Black patients (21.3 vs. 24.5 months, *p* < 0.001) [35]. Thus, further work is needed to understand the effects of these treatments in other datasets that may have a larger proportion of Black patients.

When assessing OS in subgroups defined by subsequent treatments, the largest subgroup of patients (~41%) received 1L NHT only. Notably, abiraterone was associated with a significantly shorter median OS versus enzalutamide in this subgroup (10.6 vs. 13.6 months, *p* = 0.001). This is consistent with the VHA and Flatiron studies showing that approximately 50% of patients with mCRPC received only one line of NHT; and abiraterone was associated with significantly reduced OS compared with enzalutamide [7–9]. While these are considered post-baseline subgroups and no adjustment for time-varying covariates was performed, the consistency of these results across datasets suggests that a significant number of patients with mCRPC will only receive one NHT and the choice of 1L NHT may have significant survival implications. The observed treatment patterns in this study were consistent with previously published real-world studies in the US [7–9, 36].

This study has some limitations. As this was a retrospective cohort study of the Medicare population, findings may not be applicable to the general population. Given the lack of specific diagnosis codes for mCRPC, assumptions were made in selecting patients with mCRPC based on clinical input and initiation of treatment with abiraterone and enzalutamide, during a period when both were approved for mCRPC only and before public disclosure of the abiraterone clinical trial findings in mCSPC. Furthermore, this study may be limited by residual confounding as we were unable to adjust survival outcomes by several potentially confounding clinical factors (e.g., performance status, laboratory measurements, tumor grade, and metastatic disease burden) due to their unavailability in the Medicare data. Our analysis utilized Medicare claim data which are inherently subject to inaccuracies in the coding of diagnoses and therapies. In addition, claims data do not provide information about the causes of change in treatment. However, as inaccuracies in data tend to bias the results to the null, it is possible our study misestimated the survival detriment associated with abiraterone. Variables such as time from first PC diagnosis to metastatic diagnosis and time from ADT to index date may be truncated because claims data were available after patients became eligible and were enrolled in Medicare. Finally, a filled pharmacy claim does not guarantee that the patient used the prescribed treatment.

In conclusion, in the Medicare chemotherapy-naïve mCRPC population, patients initiating 1L abiraterone had significantly shorter survival and increased risk of death compared with patients initiating 1L enzalutamide. Abiraterone-associated survival detriments were observed in patients with older age, White patients, low and middle/high SES, and certain comorbidities. These findings support previous real-world studies of large databases reporting worse OS associated with 1L abiraterone versus enzalutamide in this patient population. The reproducibility of these results across varied populations represents mounting evidence of a significant difference in comparative effectiveness among the two NHTs. Given the significant proportion of patients who ultimately receive only one line of therapy for mCRPC and the lack of any subgroups demonstrating improved survival with abiraterone, these data should support greater use of enzalutamide in this patient population. Future studies utilizing different data sources to fully determine the impact of 1L abiraterone compared with enzalutamide on patient survival in mCRPC should ensure an appropriate balance between statistical power and the ability to detect clinically meaningful differences, as null findings seem to be exclusively found in smaller studies that were likely underpowered.

## Supplementary information

Supplementary Information

## Data Availability

The data that support the findings of this study are available from Medicare, but restrictions apply to the availability of these data, which were used under license for the current study, and so are not publicly available.

## Code availability

The code cannot be made publicly available due to confidentiality and proprietary rights. The macros, methods, and model specifics can be shared upon request.
