# Supplementary material for: Real-world overall survival with abiraterone acetate versus enzalutamide in chemotherapy-naïve patients with metastatic castration-resistant prostate cancer
Source: Prostate Cancer Prostatic Dis. 2024 Mar 27;27(4):756–64. doi: 10.1038/s41391-024-00816-0 (PMC11543594; doi:10.1038/s41391-024-00816-0)
Supplement: Supplementary file 1 — Supplementary Information [file 41391_2024_816_MOESM1_ESM.docx]

# Supplementary Information

## Supplemental methods

The data contain patient demographics, including race, enrollment, and claims history; including drug, diagnosis, physician visits, and procedures (Medicare Part A, B, and D). The dates of death in the data were verified against records from the US Social Security Administration agency or the Railroad Retirement Board [1].

### Study power and sample size

Study power was assessed using a Cox proportional-hazards regression analysis for nonbinary covariates [2], assuming a hazard ratio (HR) of 1.19 for overall survival between abiraterone and enzalutamide (as the reference group) based on Tagawa et al [3], a two-sided alpha-level of 5%, an R-squared of treatment cohort and covariates of 0.25, and different estimates for the percentage of patients in each treatment cohort and the percentage of deaths during the follow-up period. With a sample size of ~5000 patients, the study power was expected to be above 90%.

### Definition of baseline characteristics

Cardiovascular disease (CVD): included major adverse cardiac events, i.e., acute myocardial infarction, stroke, unstable angina or revascularization procedures, or heart failure (cardiovascular death excluded), as well as peripheral arterial disease, venous thromboembolism, arrhythmia, and hypertensive emergency/urgency/crisis unspecified.

Geographic regions: patients that lived in Northeast, Midwest, South, West areas, and other/unknown.

Long-term corticosteroid use: defined as having one of the following:
1) Continuous use for at least 90 days without a gap of more than 30 days, between consecutive pharmacy claims (per Medicare Part D data), during the baseline period. 2) At least two corticosteroid procedure claims (per Medicare Part B data) with at least 90 days apart during the baseline period.

Modified Charlson Comorbidity Index (CCI): the National Cancer Institute (NCI) version of the CCI was created during the baseline period. Binary variables (yes/no) were created to flag patients who had claims for each of the individual comorbidities using the NCI codes for defining comorbidities in International Classification of Disease (ICD)-9-CM and ICD-10 administrative data (**Table S1**). Cancers were excluded from CCI.

Socioeconomic status (SES): low SES was defined based on having Medicaid dual enrollment status or eligibility for the Medicare Part D low-income subsidy [4]; the remaining patients who did not fulfill the criteria for low SES were defined as middle/high SES.

### Supplemental statistical analysis

Analyses were conducted using SAS Enterprise Guide version 7.1 (SAS Institute, Cary, North Carolina, USA). Sankey diagrams were created using R 3.6.3.

#### Inverse probability treatment-weighting (IPTW)

For comparing time-to-event outcomes between index treatment cohort analysis, IPTW was used to balance patient characteristics. Patients’ weights were a function of the propensity score: weights for abiraterone patients were 1 / (probability of initiating abiraterone), whereas weights for enzalutamide patients were 1 / (1 - probability of initiating abiraterone). To address the possibility of extreme weight values, patients’ weights were stabilized by the marginal probability of being in their treatment group. The probability of a patient receiving abiraterone compared with enzalutamide conditional on the observed baseline covariates (e.g., age, gender, comorbidities, etc.) was calculated from the logistic model. The “stabilized” IPTW weight for individual i in treatment cohort j was calculated as the inverse of the probability of receiving the corresponding treatment multiplied by the mean probability across all patients in that treatment cohort:

$$Weight_{i}=\frac{1}{Probability_{i}}\times\frac{\sum_{i=1}^{N_{j}} Pro{bability}_{i}}{N_{j}}$$

where N_j_ is the sample size of treatment cohort j.

#### Propensity score

Propensity scores for IPTW were generated based on a logistic regression model that included treatment group (abiraterone or enzalutamide; enzalutamide as the reference cohort) as the dependent variable, and the following baseline characteristics as the covariates: age, race, geographic regions, SES, site of metastasis, liver metastasis, time from diagnosis to metastasis, time from metastasis to index date, time from androgen deprivation therapy (ADT) start to index date, radical prostatectomy, prior first-generation antiandrogens, prior long-term corticosteroid use, opioid analgesic use, select baseline comorbidities (modified CCI components, type I and type II diabetes, CVD, and anemia), PC-related hospitalization, PC-related emergency room (ER) visit, all-cause hospitalization, and all-cause ER visits.

#### Sensitivity analysis

In the sensitivity analysis, overall survival (OS) between abiraterone and enzalutamide cohorts was compared among the IPTW-adjusted sample, with propensity scores estimated based on a logistic regression model adjusting for another set of covariates. Compared with the logistic regression model in the primary analysis, the sensitivity analysis model additionally adjusted for prior use of ketoconazole and diagnoses of hypertension and obesity during the baseline period.

Table S1. Administrative codes for comorbidities.

| **Conditions and comorbidities** | **ICD-9-CM** | **ICD-10-CM** |
| --- | --- | --- |
| **NCI comorbidity index^a^** |  |  |
| Cerebrovascular disease | 362.34, 430–438 | G45, G46, H34.0, I60–I69 |
| Chronic pulmonary disease | 416.8, 416.9, 490–505, 506.4, 508.1, 508.8 | I27.8, I27.9, J40–J47, J60–J67, J68.4, J70.1, J70.3 |
| Congestive heart failure | 398.91, 402.01, 402.11, 402.91, 404.01, 404.03, 404.11, 404.13, 404.91, 404.93, 425.4–425.9, 428 | I09.9, I11.0, I13.0, I13.2, I25.5, I42.0, I42.5–I42.9, I43, I50, P29.0 |
| Dementia | 290, 294.1, 331.2 | F00–F03, F05.1, G30, G31.1 |
| Diabetes with chronic complication | 250.4–250.7 | E10.2–E10.5, E10.7, E11.2–E11.5, E11.7, E12.2–E12.5, E12.7, E13.2–E13.5, E13.7, E14.2–E14.5, E14.7 |
| Diabetes without chronic complication | 250.0–250.3, 250.8, 250.9 | E10.0, E10.1, E10.6, E10.8, E10.9, E11.0, E11.1, E11.6, E11.8, E11.9, E12.0, E12.1, E12.6, E12.8, E12.9, E13.0, E13.1, E13.6, E13.8, E13.9, E14.0, E14.1, E14.6, E14.8, E14.9 |
| Hemiplegia or paraplegia | 334.1, 342, 343, 344.0–344.6, 344.9 | G04.1, G11.4, G80.1, G80.2, G81, G82, G83.0–G83.4, G83.9 |
| Mild liver disease | 070.22, 070.23, 070.32, 070.33, 070.44, 070.54, 070.6, 070.9, 570, 571, 573.3, 573.4, 573.8, 573.9, V42.7 | B18, K70.0–K70.3, K70.9, K71.3–K71.5, K71.7, K73, K74, K76.0, K76.2–K76.4, K76.8, K76.9, Z94.4 |
| Moderate or severe liver disease | 456.0–456.2, 572.2–572.8 | I85.0, I85.9, I86.4, I98.2, K70.4, K71.1, K72.1, K72.9, K76.5, K76.6, K76.7 |
| Peripheral vascular disease | 093.0, 437.3, 440, 441, 443.1–443.9, 447.1, 557.1, 557.9, V43.4 | I70, I71, I73.1, I73.8, I73.9, I77.1, I79.0, I79.2, K55.1, K55.8, K55.9, Z95.8, Z95.9 |
| Renal disease | 403.01, 403.11, 403.91, 404.02, 404.03, 404.12, 404.13, 404.92, 404.93, 582, 583.0–583.7, 585, 586, 588.0, V42.0, V45.1, V56 | I12.0, I13.1, N03.2–N03.7, N05.2–N05.7, N18, N19, N25.0, Z49.0–Z49.2, Z94.0, Z99.2 |
| **Individual comorbidities** |  |  |
| Hypertension | 362.11, 401.xx–405.xx, 437.2 | H35.039, I10–I13, I15–I16, I67.4 |
| Stroke | 430–434, 436, 362.31–362.34 | I60, I61, I62, I64, I65, I67, H34.1, H34.23, H34.21, H34.0 |
| Angina pectoris | 413 | I20 |
| Arrhythmia | 427.1, 427.4, 427.41, 427.42, 427.5, 427.69, 427.2, 427.60, 427.8, 427.89, 427.9 | I47.0, I47.2, I49.0, I46, I49.3, I49.49, I47.9, I49.40, I49.5, I49.8, I49.9 |
| Myocardial infarction | 410, 412 | I21, I22, I25.2 |
| Acute coronary syndrome | 411.x, 410.xx | I24, I21 |
| Congestive heart failure | 428.xx | I09.9, I11.0, I13.0, I13.2, I25.5, I42.0, I42.5–I42.9, I43.x, I50.x, P29.0 |
| Hyperlipidemia | 272.0–272.4 | E78.00, E78.01, E78.1, E78.2, E78.3, E78.41, E78.49, E78.5 |
| Lower-extremity arterial occlusive disease | 444.22 | I74.3, I74.4 |
| Type II diabetes | 250.0–250.3, 250.7 | E10.0, E10.1, E10.6, E10.8, E10.9, E11.0, E11.1, E11.6, E11.8, E11.9, E12.0, E12.1, E12.6, E12.8, E12.9, E13.0, E13.1, E13.6, E13.8, E13.9, E14.0, E14.1, E14.6, E14.8, E14.9 |
| Chronic obstructive pulmonary disease | 490–492, 494, 496 | J40, J41, J42, J43, J47, J44.9 |
| Impotence | 607.84, 302.72 | F52.21, N52 |
| Rheumatologic conditions | 446.5, 714.8, 710.0–710.4, 714.0–714.2 | M05, M06, M31.5, M32, M33, M34, M35.1, M35.3, M36.0 |
| Peptic ulcer disease | 531, 532, 533 | K25, K26, K27, K28 |
| Inflammatory bowel disease |  |  |
| Crohn’s disease | 555 | K50 |
| Ulcerative colitis | 556 | K51 |
| Anemia | 280–283, 284.81, 285 | D50–D53, D59–D64 |
| Seizure | 345.xx, 780.3 | G40, R56 |
| Obesity | 278.00, 278.03, 278.01, V85.3, V85.41–V85.45 | E66.01, E66.09, E66.1, E66.2, E66.8, E66.9, Z68.31–Z68.39, Z68.41–Z68.45, Z68.30 |

*ICD-9-CM* International Classification of Diseases Ninth Revision Clinical Modification, *ICD-10-CM* International Classification of Diseases Tenth Revision Clinical Modification, *NCI* National Cancer Institute.

^a^Adapted using codes for defining comorbidities in ICD-9-CM and ICD-10 administrative data [5] available in NCI Comorbidity Index Overview: <https://healthcaredelivery.cancer.gov/seermedicare/considerations/comorbidity.html>

Table S2. Additional baseline demographics and clinical characteristics.

|  | **Treatment cohort** | | |
| --- | --- | --- | --- |
|  | **Abiraterone (*n* = 2911)** | **Enzalutamide (*n* = 2595)** | **Standardized difference^a^ (%)** |
| **Demographics at index date^b^** |  | | |
| Age categories (years), *n* (%) |  | | |
| ≤ 64 | 91 (3.1) | 84 (3.2) | -0.63 |
| 65‒69 | 371 (12.7) | 336 (12.9) | -0.61 |
| 70‒74 | 530 (18.2) | 485 (18.7) | -1.25 |
| 75‒79 | 649 (22.3) | 548 (21.1) | 2.86 |
| 80‒84 | 627 (21.5) | 514 (19.8) | 4.28 |
| ≥ 85 | 643 (22.1) | 628 (24.2) | -5.01 |
| Geographic regions, *n* (%) |  | | |
| South | 1076 (37.0) | 937 (36.1) | 1.78 |
| Midwest | 711 (24.4) | 620 (23.9) | 1.24 |
| West | 581 (20.0) | 513 (19.8) | 0.48 |
| Northeast | < 550 (20.0) | < 550 (20.0) | -4.52 |
| Other^c^ | < 11 (0.4) | < 11 (0.4) | – |
| Unknown | < 11 (0.4) | < 11 (0.4) | – |
| **Clinical characteristics** |  |  |  |
| Time from PC diagnosis date to metastatic disease diagnosis (months)^d^, median (IQR) | 22.8 (0.9–58.6) | 24.1 (1.2–63.4) | -5.09 |
| Time from metastatic disease diagnosis to index date (months)^d^, median (IQR) | 11.6 (2.9–25.1) | 11.2 (1.6–25.8) | 1.55 |
| Time from first ADT to index date (months)^e^, median (IQR) | 29.4 (14.1–58.7) | 31.4 (14.9–60.0) | -4.48 |
| Medication and procedure history, *n* (%) |  |  |  |
| Opioid analgesics | 1577 (54.2) | 1348 (51.9) | 4.47 |
| First-generation antiandrogens |  |  |  |
| Bicalutamide | 1981 (68.1) | 1688 (65.0) | 6.37 |
| Flutamide | 37 (1.3) | 41 (1.6) | -2.61 |
| Nilutamide | 23 (0.8) | 31 (1.2) | -4.08 |
| Individual comorbidities^f^, *n* (%) |  |  |  |
| Cerebrovascular disease | 496 (17.0) | 473 (18.2) | -3.12 |
| Arrhythmia | 524 (18.0) | 443 (17.1) | 2.44 |
| Obesity | 381 (13.1) | 385 (14.8) | -5.04 |
| Stroke | 357 (12.3) | 354 (13.6) | -4.10 |
| Any liver disease | 325 (11.2) | 289 (11.1) | 0.09 |
| Myocardial infarction | 317 (10.9) | 256 (9.9) | 3.36 |
| Impotence | 214 (7.4) | 223 (8.6) | -4.59 |
| Acute coronary syndrome | 151 (5.2) | 132 (5.1) | 0.46 |
| Dementia | 141 (4.8) | 132 (5.1) | -1.12 |
| Angina pectoris | 142 (4.9) | 100 (3.9) | 5.02 |
| Rheumatologic disease | 84 (2.9) | 63 (2.4) | 2.85 |
| Seizure | 92 (3.2) | 62 (2.4) | 4.7 |
| Hemiplegia | 60 (2.1) | 64 (2.5) | -2.72 |
| Paralysis | 58 (2.0) | 62 (2.4) | -2.71 |
| Peptic ulcer disease | 52 (1.8) | 52 (2.0) | -1.6 |
| Inflammatory bowel disease | 31 (1.1) | 19 (0.7) | 3.53 |
| Ulcerative colitis | 25 (0.9) | 14 (0.5) | 3.83 |
| Crohn's disease | < 11 (0.4) | < 11 (0.4) | 2.33 |
| Lower-extremity arterial occlusive disease | 15 (0.5) | 14 (0.5) | -0.33 |

*ICD* International Classification of Diseases.

^a^The standardized difference was multiplied by 100 to get the percent standardized difference. A value > 10% or < -10% is considered a significant imbalance..

^b^The index date was defined as the first initiation of abiraterone or enzalutamide within 90 days prior to or any time after a metastatic disease diagnosis following prostate cancer diagnosis, and during the index period of September 10, 2014 through May 31, 2017.

^c^Other regions include Puerto Rico, the Virgin Islands, Canada, Mexico, Central America and West Indies, Europe, Northern Marianas, and Guam.

^d^First observed metastatic disease diagnosis after the PC diagnosis present in the data was used. For patients who reached metastatic disease after index date, their time from metastatic disease to index date was set to 0.

^e^Date of first ADT was defined by the earliest of surgical castration or medical castration during any time prior to the index date.

^f^See Table S1 for a full list of ICD codes used to identify individual comorbidities.

Fig. S1 Sample selection of patients with mCRPC.


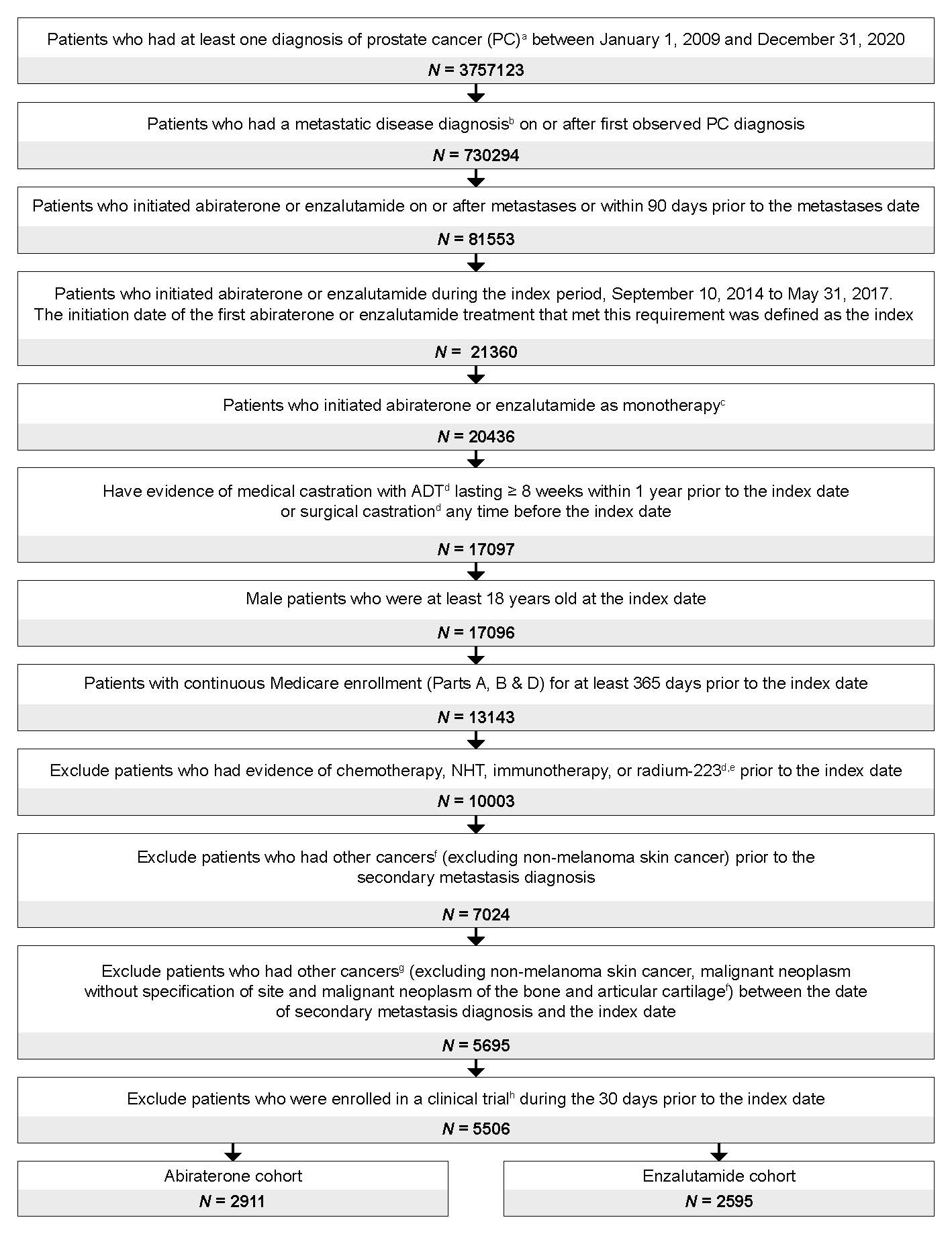


*ADT* androgen deprivation therapy*, ICD* International Classification of Diseases*, ICD-9-CM* ICD-Ninth Revision-Clinical Modification*, ICD-10-CM* ICD-Tenth Revision-Clinical Modification*, mCRPC* metastatic castration-resistant prostate cancer*, NHT* novel hormonal therapy*, PC* prostate cancer*.*

^a^Diagnosis of PC was identified using ICD-9-CM diagnosis code (185) and ICD-10-CM diagnosis code (C61).

^b^Metastatic disease was identified using ICD-9-CM diagnosis codes (196–199.0) and ICD-10-CM diagnosis codes (C77, C78, C79, C80.0, and C7B).

^c^Patients receiving another NHT, chemotherapy, immunotherapy, radium-223, ketoconazole, olaparib, and rucaparib on the index date or within the 28 days following the index date were excluded.

^d^ADT, surgical and medical castration, chemotherapy, NHT, immunotherapy, and radium-223 were identified using administrative codes.

^e^Patients were excluded for evidence of chemotherapy and/or NHT use at any time prior to the index date. The exclusion window for immunotherapy and radium-223 was during the baseline period (12 months prior to index date).

^f^Other cancers were identified using ICD-9-CM diagnosis codes (140–172, 174–184, 186–195, 199.1, 199.2, and 200–209) and ICD-10-CM diagnosis codes (C00–C43, C45–C60, C62, C63, C76, C80.1, C80.2, C81–C96, and C7A).

^g^Malignant neoplasm without specification of site was identified using ICD-9-CM diagnosis codes (199.1 and 199.2) and ICD-10-CM diagnosis codes (C80.1 and C80.2). Malignant neoplasm of the bone and articular cartilage was identified using ICD-9-CM diagnosis code (170) and ICD-10-CM diagnosis codes (C40–C41).

^h^Clinical trial participation was identified using ICD-9-CM diagnosis code (V70.7) and ICD-10-CM diagnosis code (Z00.6).

Fig. S2 IPTW-adjusted pairwise OS comparison in the overall population of patients with chemotherapy- and NHT-naïve mCRPC after adjusting for additional covariates.


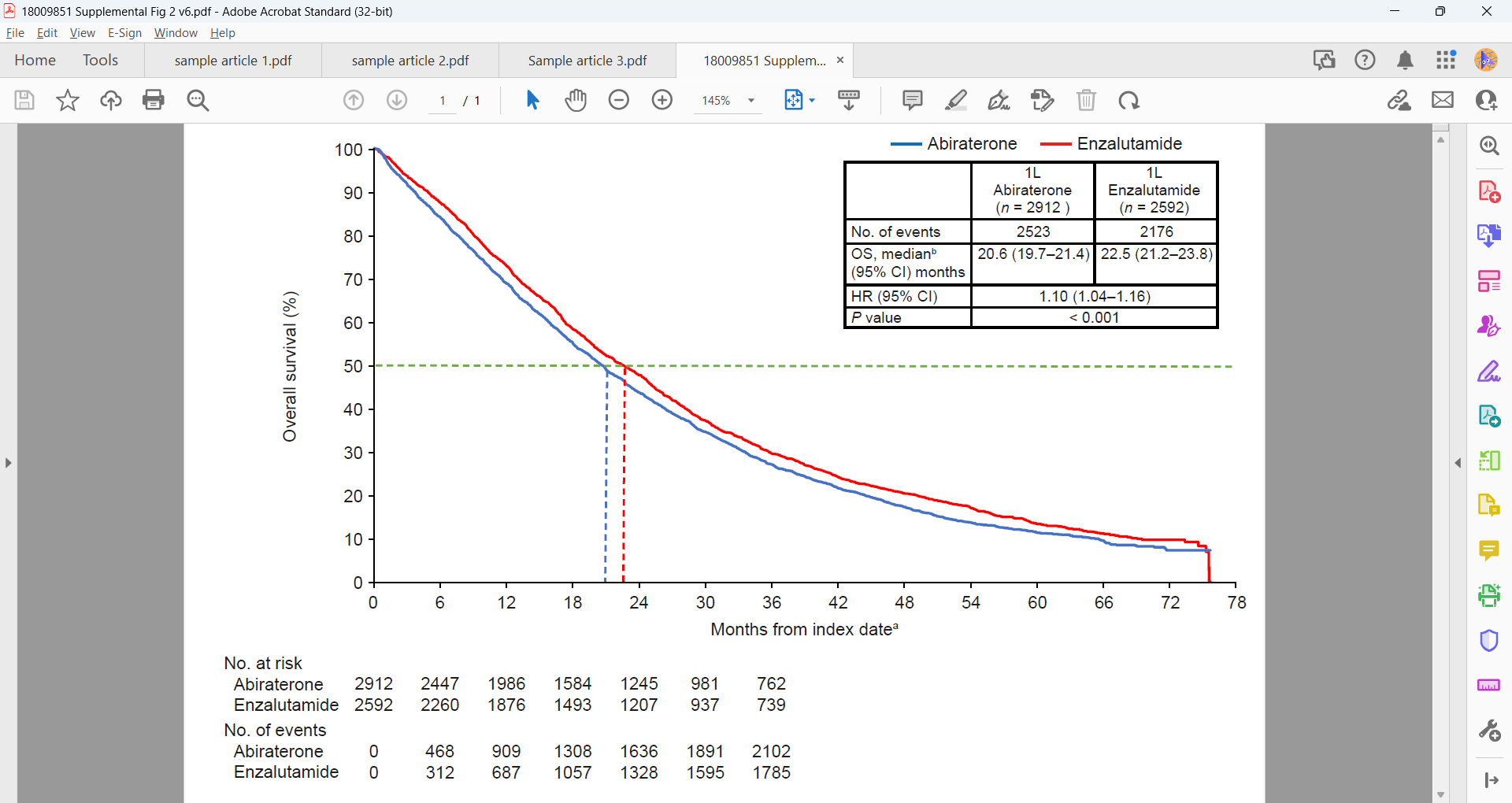


*1L* first-line, *ADT* androgen deprivation therapy, *CCI* Charlson Comorbidity Index, *CI* confidence interval, *HR* hazard ratio, *IPTW* inverse probability treatment-weighting, *mCRPC* metastatic castration-resistant prostate cancer, *NHT* novel hormonal therapy, *OS* overall survival, *PC* prostate cancer, *SES* socioeconomic status.

^a^The index date was defined as the first initiation of abiraterone or enzalutamide within 90 days prior to or any time after a metastatic disease diagnosis following prostate cancer diagnosis, and during the index period of September 10, 2014 through May 31, 2017.

^b^Median OS represents the IPTW-weighted OS among patients with mCRPC treated with 1L abiraterone or enzalutamide therapy during the entire follow-up period, adjusting for another set of covariates. Propensity scores for IPTW were generated by adjusting for baseline characteristics including age, race, geographic regions, index year, SES, site of metastasis, liver metastasis, time from diagnosis to metastasis, time from metastasis to index date, time from ADT start to index date, radical prostatectomy, prior first-generation antiandrogens, prior ketoconazole, prior chronic corticosteroid use, opioid analgesic use, comorbidities during baseline (modified CCI components, type I and type II diabetes, cardiovascular disease, anemia, hypertension, and obesity), PC-related hospitalization, PC-related emergency room visit, all-cause hospitalization, and all-cause emergency room visits.

# Supplementary References

1. Research Data Assistance Center. Data Documentation: Master Beneficiary Summary File Base. 2019. <https://resdac.org/cms-data/files/mbsf-base/data-documentation>.

2. Hsieh FY, Lavori PW. Sample-size calculations for the Cox proportional hazards regression model with nonbinary covariates. Control Clin Trials. 2000;21:552-560.

3. Tagawa ST, Ramaswamy K, Huang A, Mardekian J, Schultz NM, Wang L, et al. Survival outcomes in patients with chemotherapy-naive metastatic castration-resistant prostate cancer treated with enzalutamide or abiraterone acetate. Prostate Cancer Prostatic Dis. 2021;24:1032-1040.

4. Centers for Medicare & Medicaid Services. Defining Medicare-Medicaid Dually Enrolled Beneficiaries in CMS Administrative Data. 2021. <https://www.cms.gov/Medicare-Medicaid-Coordination/Medicare-and-Medicaid-Coordination/Medicare-Medicaid-Coordination-Office/Downloads/MMCO_DualEligibleDefinition.pdf>.

5. Quan H, Sundararajan V, Halfon P, Fong A, Burnand B, Luthi JC, et al. Coding algorithms for defining comorbidities in ICD-9-CM and ICD-10 administrative data. Med Care. 2005;43:1130-1139.
